# Health insurance drop-out among adult population: findings from a study in a Health and demographic surveillance system in Northern Vietnam 2006–2013

**DOI:** 10.1017/gheg.2016.14

**Published:** 2016-10-14

**Authors:** Hoang Van Minh, Tran Quynh Anh, Nguyen Thi Thuy Nga

**Affiliations:** Hanoi School of Public Health, 138 Giang Vo Street, Ba Dinh District, Hanoi, Vietnam

**Keywords:** Drop-out, health insurance, socio-economic correlates, Vietnam

## Abstract

The coverage of health insurance as measured by enrollment rates has increased significantly in Vietnam. However, maintaining health insurance to the some groups such as the farmer, the borderline poor and informal workers, etc. has been very challenging. This paper examines the situation of health insurance drop-out among the adult population in sub-rural areas of Northern Vietnam from 2006 to 2013, and analyzes several socio-economic correlates of the health insurance drop-out situation. Data used in this paper were obtained from Health and Demographic Surveillance System located in Chi Linh district, an urbanizing area, in a northern province of Vietnam. Descriptive analyses were used to describe the level and distribution of the health insurance drop-out status. Multiple logistic regressions were used to assess associations between the health insurance drop-out status and the independent variables. A total of 32 561 adults were investigated. We found that the cumulative percentage of health insurance drop-out among the study participants was 21.2%. Health insurance drop-out rates were higher among younger age groups, people with lower education, and those who worked as small trader and other informal jobs, and belonged to the non-poor households. Given the findings, further attention toward health insurance among these special populations is needed.

## Introduction

Social Health Insurance (HI) was first introduced in Vietnam in 1992 and is currently a key policy for achieving Universal Health Coverage (UHC) in Vietnam. The first policy on HI (Decree No. 299/1992) was started with a compulsory scheme (formal workers and pensioners) and voluntary scheme for the others. With the implementation of Decree No. 58/1998, the HI was expanded to people with merits. The implementation of Decree No. 63/2005 expanded the coverage of compulsory HI scheme to the poor and ethnic minorities (premium subsidies from the government budget). From 2009, when the Law on Health Insurance was introduced and based on the Decree No. 62/2009, compulsory enrollment had been introduced for children under 6, students and other remaining groups. The Vietnamese government has outlined a roadmap to Universal Health Insurance by 2014, which was clearly stated in the Health Insurance law 2008 [1]. The goal was reset by the Vietnamese Government to achieve at least 70% and 80% coverage by 2015 and 2020 respectively [2].

The coverage of HI as measured by enrollment rates has increased significantly over the years. The coverage went up from 10% in 1995 to 68.5% in 2012. However, extending HI to some groups such as farmers, the borderline poor and informal workers, etc. has been very challenging [3, 4]. Even when attempts are made to extend HI coverage for vulnerable groups, maintaining their membership becomes problematic as they are likely to drop out due to problems associated with membership renewal [5]. This poses a major threat on the transition towards UHC in developing countries [6]. Dropout from insurance enrollment also hampers resource mobilization for effective scheme management and creates long-term sustainability problems.

Given the difficulties associated with retaining health insurance membership, especially for the traditionally excluded informal sector, the need to understand reasons why people drop out from insurance enrollment becomes a relevant policy and research issue. We aimed to examine the situation of health insurance drop out among adult populations in a study of a sub-rural area in Northern Vietnam between 2006 and 2013 and analyze several socio-economic correlates of the health insurance drop out situation.

## Methods

### Data source and the study site

Data used in this paper was obtained from a Health and Demographic Surveillance System located in an urbanizing area of the Chi Linh district of Hai Duong, a northern province of Vietnam (CHILILAB HDSS). The CHILILAB HDSS collects longitudinal data on demographic and health indicators in Chi Linh district since 2004. Within the CHILILAB HDSS, 57 561 people from 17 993 households in three towns and four communes have been surveyed. As of December 2013, five rounds of a baseline survey (collecting data on the basis of socio-economic household and individual such as age, gender, education, occupation, economic status, and insurance status, etc.) and 17 periodic update surveys (collecting information on population changes such as birth, death, migration, marriage, and pregnancy, etc.) had been conducted. More information on the study sites and data collection process has been described elsewhere. Descriptive analyses were used to describe the level and distribution of the health insurance drop-out status. Multiple logistic regressions were used to assess associations between the health insurance drop-out status and the independent variables. Odds ratios (ORs) are used to assess the magnitude of associations and 95% confidence intervals (95% CI) are reported. Statistical significance was set at *p* < 0.05.

### Study variables

In CHILILAB, health insurance status were measured since 2006 and re-assessed in 2008, 2010, 2011, and 2013. The heads of household reported whether or not each of his/her family members currently enrolled in any health insurance scheme at the time of survey. In this paper, the dependent variable is health insurance drop-out practice among adult population (aged 25 years and over) the CHILILAB HDSS. Health insurance drop-out case is defined as any adult who had no health insurance in the last round of survey (2013), while he/she ever had health insurance in any of the preceding rounds of survey (i.e. 2006 or 2008 or 2010 or 2011). The independent variables were socio-economic characteristics of the study respondents at the last round of survey (2013), including: (1) Age groups (1 = 25–34, 2 = 35–44, 3 = 45–54, 4 = 55–64 and 5 = 65 years and older); (2) Gender (1 = men and 2 = women); (3) Education (1 = less than secondary school, 2 = completed secondary school, 3 = completed high school, and 4 = College/University degrees); (4) Occupation (1 = government staffs, enterprise/factory workers, 2 = farmers, and 3 = small trader and other informal jobs such as temporary construction workers, motorbike taxi drivers, etc.); and (5) Economic situation (1 = poor household and 2 = non-poor household).

### Data analyses

The data were cleaned and analyzed using Stata statistical software version 12. Cases with missing data on any independent variables were excluded from the analysis. Descriptive analyses were used to describe the level and distribution of the health insurance drop-out status. Multiple logistic regressions were used to assess associations between the health insurance drop-out status and the independent variables. Odds ratios (ORs) are used to assess the magnitude of associations and 95% confidence intervals (95% CI) are reported. Statistical significance was set at *p* < 0.05. This study was approved by Ethical Review Board of the Hanoi School of Public Health.

## Results

Table 1 describes general characteristics of the study respondents in the last round of survey (2013). A total of 32 561 adults were investigated. Most of the respondents aged <64 years at the time of the survey (12.5% of them aged 65 years old and over). There were slightly more women than men in the study sample (51.2% *v*. 48.8%, respectively). More than 90% of the study respondents had completed secondary school or higher education (9.5% had less than secondary school education). More than half worked as Government staffs and enterprise/factory workers (only 29.8% worked as farmers). The proportion of poor households (as identified by local authorities) was 3.3%.

**Table 1.** General characteristics of the study respondents in the last round of survey (2013)

Characteristics	Count	Percentage
Age groups		
25–34	7714	23.5
35–44	7729	23.5
45–54	8314	25.3
55–64	4977	15.2
65+	4094	12.5
Gender		
Men	16 010	48.8
Women	16 818	51.2
Education		
Less than secondary school	3124	9.5
Completed secondary school	3927	12.0
Completed high school	18 382	56.0
College , University degree	7395	22.5
Occupation		
Government staffs, enterprise/factory workers	16 835	51.3
Farmers	9774	29.8
Small trader and other informal jobs	6219	18.9
Economic situation		
Non-poor	31 491	96.7
Poor	1070	3.3
Total	32 561	100.0

**Fig. 1.**
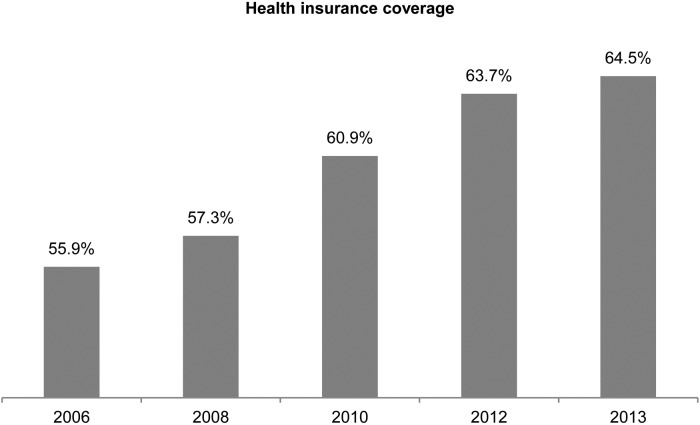
Coverage of health insurance among the study respondents during 2006–2013.

**Fig. 2.**
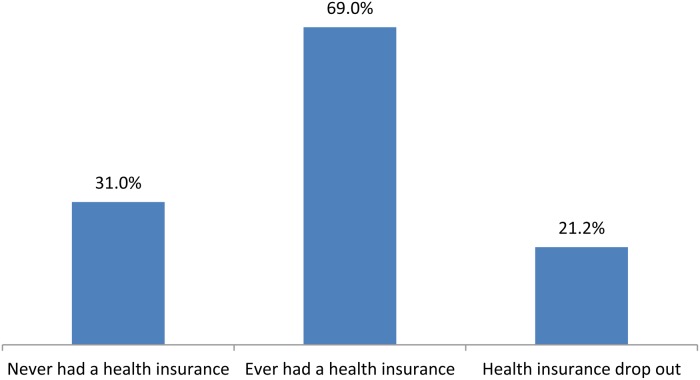
Health insurance status among the study respondents during 2006–2013.

The coverage of HI among the study respondents gradually increased over the years, from 56% in 2006 to 64.5% in 2013 (Fig. 1). During 2000–2013, while 69% of the adults had health insurance at any given time, 31% of them had never been enrolled in any health insurance scheme. The cumulative percentage of HI drop-out among the study participants over the study period was 21.2% (Fig. 2).

Table 2 presents the distributions of HI drop-out rates among the study respondents by their socio-economic statuses. The health insurance drop-out rates were higher among younger age groups (highest rate of 30.3% among those aged 35–44 years), people with lower education (highest rate of 34.% among those completed secondary school), those who worked as small traders and other informal jobs (50.7%), and the non-poor (22.2%).

**Table 2.** Distributions of health insurance drop-out rates among the study respondents by their socio-economic statuses

Characteristics	Count	Percentage
Age groups		
25–34	1160	24.1
35–44	1195	30.3
45–54	1248	21.9
55–64	730	16.6
65+	471	12.4
Gender		
Men	2321	21.0
Women	2483	21.5
Education		
Less than secondary school	488	18.8
Completed secondary school	782	34.0
Completed high school	3153	29.4
College, University	381	5.4
Occupation		
Government staffs, enterprise/factory workers	1332	9.1
Farmers	2051	39.1
Small trader and other informal jobs	1421	50.7
Economic situation		
Non-poor	4735	22.2
Poor	50	4.9
Total	4785	21.4

Table 3 reports ORs and their 95% CI from the multiple logistic regression analysis of the association between health insurance drop-out status among the study respondents and their socio-economic statuses. We found that, after holding all other independent variables constant, statistically significant correlates of being HI dropout case were: (1) Younger age: the highest odds of being a health insurance dropout case was found among people aged 25–34 compared with those aged 65 years older or older (OR of 4.2, 95% CI of 3.5–4.9); (2) Lower education level: the highest odds of being a health insurance dropout case was found among respondents who had less than secondary school (OR of 7.8, 95% CI of 6.5–8.3); (3) Working as farmer or small trader or informal workers: the highest odds of being health insurance dropout case was found among small trader and other informal jobs (OR of 7.8, 95% CI of 7.1–8.7); (4) Economic status: the non-poor were more likely to be health insurance dropout case than the poor (OR of 13.9, 95% CI of 10.4–18.8).

**Table 3.** Multiple logistic regressions analysis of the association between health insurance drop-out status among the study respondents and their socio-economic statuses

Characteristics	Odds ratio	95% CI	
		Lower bound	Upper bound
Age groups			
25–34	4.2*	3.7	4.9
35–44	2.8*	2.4	3.3
45–54	1.6*	1.4	1.9
55–64	1.1	0.9	1.3
65+	1		
Gender			
Men	1		
Women	0.8	0.7	1.8
Education			
Less than secondary school	7.8*	6.5	9.3
Completed secondary school	7.0*	6.0	8.2
Completed high school	4.4*	3.9	5.0
College, University	1		
Occupation			
Government staffs, enterprise/factory workers	1		
Farmers	5.5*	5.0	6.0
Small trader and other informal jobs	7.8*	7.1	8.7
Economic situation			
Non-poor	13.9*	10.4	18.8
Poor	1		
Total			

*Denotes significant findings.

## Discussion

Little is known about situation of HI drop out and its socio-economic correlates in Vietnam. We found the cumulative percentage of health insurance drop-out among the study participants during 2006–2013 was 21.2%. Unaffordability of premium and adverse selection especially in the voluntary group are considered as the main causes of this reduction in this study. Notably, since there are no conditions on minimum enrollment percentage for voluntary scheme from 2008 to 2014, there was a high risk of adverse selection [4]. The poor quality of health care service for HI cardholders is also a possible reason for dropout. There has been consensus that enrollment decisions are linked to trust for services offered by healthcare providers. Trust is generated by clients’ previous experience of quality of care, and health care providers’ ability to offer services that meet their expectations during service use [7]. Perception of poor quality of health services is identified as the most important determinant of dropout [8]. Limited benefits as well as failure to provide promised benefits can negatively affect the decision to remain insured [9].

A study conducted in Ghana reported that the proportion of health dropouts increased from 6.8% in 2008 to 34.8% in 2012. The study found similar reasons for dropping out, including unaffordability of the premium, followed by the scheme's limited benefits for rare illnesses and poor service quality; cost of premium was less likely a reason for dropout by all the different age subgroups [10]. Another study by Sommers in the USA in 2009 found that, each year, about 2 million adults left Medicaid and became uninsured. Disenrollment was significantly higher among adults than children [11].

Regarding socio-economic correlates of the health insurance dropout situation, our multiple logistic regression analyses revealed that the significant correlates of being health insurance dropout case include (1) younger age, (2) lower education levels, (3) informal job, and (4) those being non-poor. Before 2014, under the Vietnamese HI regulations, the informal and non-poor workers were under the voluntary, contributory subcategory. The HI enrollment of this informal group was the lowest coverage of 26% in 2011 [3]. These findings are both similar and different from those reported by previous studies. Hendryx *et al*. demonstrated that persons who dis-enrolled were more likely to be younger adults [6]. A study from Bukina Faso found that higher age or lower education of a household head was associated with a higher rate of insurance drop-out [5]. A study from Ghana also reported that, compared with respondents with primary/junior high education, access to senior high education or higher significantly reduced the likelihood of attributing dropout of the scheme to cost of premium [10].

In terms of economic status, the above mentioned study from Ghana revealed that the cost of premium had less significant influence on dropout by respondents engaged in the formal sector and informal sector compared with the unemployed. Okeke *et al*. found that lower-paid workers are disproportionately more likely to drop coverage than higher-paid workers [12]. The cost of premium was less likely to be a cause of dropout by those within the high-income cohort, it highly influenced drop-out decisions by those in the low-income category [10]. Wagstaff observed that individuals are unlikely to insure as they move closer to poverty because any decrease in income can push them further towards mere survival [13]. Premium increases and co-payments in Oregon's Medicaid program were found to be the main driver of disenrollment, followed by benefit elimination [14]. Similarly, higher household expenditure was shown to be correlated with higher HI drop-out rates [5, 7, 15]. Similarly, as a results of increased cost sharing revealed three main reasons for disenrollment, which varied by enrollees’ incomes, including: finding other coverage, becoming financially ineligible, or dropping coverage as too expensive [6].

## Conclusion

We found that the cumulative percentage of health insurance drop-out among the study participants was high. Health insurance drop-out rates were higher among younger age groups, people with lower education, those who worked as small trader and other informal jobs, and belonged to the non-poor households. Given the findings, further attentions toward health insurance among these special groups of population are needed.
